# Role and targeting of anaplastic lymphoma kinase in cancer

**DOI:** 10.1186/s12943-018-0776-2

**Published:** 2018-02-19

**Authors:** Carminia Maria Della Corte, Giuseppe Viscardi, Raimondo Di Liello, Morena Fasano, Erika Martinelli, Teresa Troiani, Fortunato Ciardiello, Floriana Morgillo

**Affiliations:** Medical Oncology, Department of Experimental and Internal Medicine “F. Magrassi”, University of Campania “Luigi Vanvitelli”, via S. Pansini 5, 80131 Naples, Italy

**Keywords:** ALK, Crizotinib, Alectinib, Ceritinib, Tyrosine kinase inhibitor, Resistance

## Abstract

Anaplastic lymphoma kinase (ALK) gene activation is involved in the carcinogenesis process of several human cancers such as anaplastic large cell lymphoma, lung cancer, inflammatory myofibroblastic tumors and neuroblastoma, as a consequence of fusion with other oncogenes (NPM, EML4, TIM, etc) or gene amplification, mutation or protein overexpression.

ALK is a transmembrane tyrosine kinase receptor that, upon ligand binding to its extracellular domain, undergoes dimerization and subsequent autophosphorylation of the intracellular kinase domain. When activated in cancer it represents a target for specific inhibitors, such as crizotinib, ceritinib, alectinib etc. which use has demonstrated significant effectiveness in ALK-positive patients, in particular ALK-positive non- small cell lung cancer.

Several mechanisms of resistance to these inhibitors have been described and new strategies are underway to overcome the limitations of current ALK inhibitors.

## Background

**Fig. 1:**
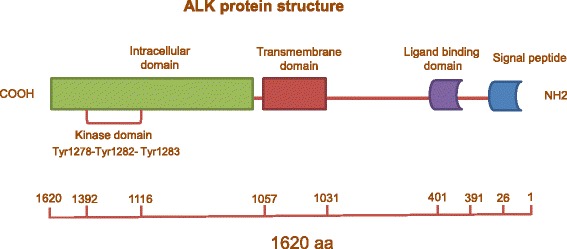
Structure of ALK protein. The human ALK protein is a polypeptid of 1620 amino acid. The ALK mature protein is a classical receptor tyrosine kinase that comprises an extracellular ligand-binding domain, a transmembrane domain, and an intracellular tyrosine kinase domain which harbors the 3-tyrosine motif (Tyr1278, Tyr1282 and Tyr1283) which represents the major auto-phosphorylation site regulating kinase activity

Anaplastic lymphoma kinase (ALK) is a receptor tyrosine kinase belonging to the insulin receptor superfamily sharing a high degree of homology with leukocyte tyrosine kinase (LTK) [1]. The human *ALK* gene is located on the 2p23 chromosomal segment and encodes for a polypeptid of 1620 amino acid which undergoes to post-translational modifications generating a mature ALK protein of approximately 200–220 kDa [2, 3]. The ALK mature protein is a classical receptor tyrosine kinase that comprises an extracellular ligand-binding domain of 1030 aminoacids (aa), a transmembrane domain (28 aa), and an intracellular tyrosine kinase domain (561 aa) [4]. The kinase domain shares with the other kinases of the same family the 3-tyrosine motif (Tyr1278, Tyr1282 and Tyr1283) which is located in the activation loop and represent the major auto-phosphorylation site of kinase activity [5, 6] (Fig. 1). ALK becomes activated only upon ligand-induced homo-dimerization, and inactivated through de-phosphorylation by receptor protein tyrosine phosphatase beta and zeta complex (PTPRB/PTPRZ1) in the absence of the ligand [7]. Two proteins, midkine and pleiotrophin, have been reported to be activating ligands for mammalian ALK [8], although they are not specific for ALK [9].

**Fig. 2:**
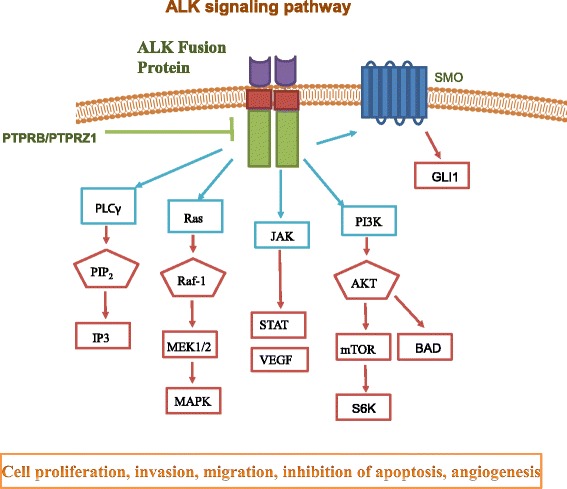
ALK signaling pathway. ALK activates multiple pathways, including phospholipase C γ, Janus kinase (JAK)-signal transducer and activator of transcription (STAT), PI3K-AKT, mTOR, sonic hedgehog (SMO and GLI), and MAPK signaling cascades, which affect cell growth, transformation and anti-apoptotic signaling. Receptor protein tyrosine phosphatase beta and zeta complex (PTPRB/PTPRZ1) inactivates ALK through de-phosphorylation

ALK activates multiple pathways, including phospholipase C γ, Janus kinase (JAK)-signal transducer and activator of transcription (STAT), Phosphoinositide 3-kinase (PI3K)-AKT, mammalian target of rapamycin (mTOR), sonic hedgehog, JUNB, CRKL-C3G (also known as RAPGEF1)-RAP1 GTPase and mitogen-activated protein kinase (MAPK) signaling cascades, which affect cell growth, transformation and anti-apoptotic signaling [9] (Fig. 2).

ALK is highly conserved across species. ALK mRNA expression is present [10] in the adult human brain, where it is thought to play a role in the development and function of the nervous system, and it is also expressed in small intestine, testis, prostate, and colon whereas human lymphoid tissues and cells, lung and other organs are excluded.

The first identification of ALK occurred in anaplastic large cell lymphoma (ALCL) as the product of a gene rearrangement [10, 11]. Since then, *ALK* rearrangement, mutations, or amplification was discovered in a series of tumors including lymphoma, neuroblastoma, and non-small cell lung cancer (NSCLC) [12].

So far, 21 different genes have been described as being translocated with *ALK* and, in addition to this complexity, within the different *ALK* fusion there are several breakpoint variants. Different *ALK* fusion proteins may be responsible for different proliferation rates, colony formation, invasion and tumorigenicity capabilities, leading to activation of various signaling pathways.

The 70–80% of all ALK-positive ALCL patients present the *ALK* gene (2p23) and the *NPM* (nucleolar phosphoprotein B23, numatrin) gene (5q35) translocation [13] with several t(2;5) breakpoint variants described. NPM (also known as NPM1), encodes for a protein which is involved in the regulation of cell division, DNA repair, transcription and genomic stability [14]. The NPM-ALK chimeric protein is constitutively expressed from the NPM promoter, leading to the overexpression of the ALK catalytic domain. Many other rearrangements involving the *ALK* gene have recently been shown to be associated with ALCL, including *ALO17-ALK, TRK-fused gene (TFG)-ALK, moesin (MSN)-ALK, Tropomyosin 3* (*TPM3)-ALK, Tropomyosin 4* (*TPM4)-ALK, ATIC-ALK, myosin 9(MYH9)-ALK, CLTC-ALK* [15]. Of interest, the chimeric protein seems to behave as neo-antigent leading to the production of autologous antibodies against chimeric protein, suggesting an immune response to the ALK protein [16].

The t(2;17)(p23;q23) translocation, which generates CLTC-ALK is also found in diffuse large B-cell lymphoma (DLBCL) and represents the most frequent chromosomal rearrangement in this disease. A small portion (0.5–1%) of DLBCLs display the NPM-ALK fusion protein or other fusion proteins such as Sequestosome 1 (SQSTM1)-ALK and SEC31A-ALK.

Inflammatory myofibroblastic tumors (IMT) were the first solid tumor to be associated with *ALK* translocation. Approximately 50% of IMT display clonal rearrangements of *ALK* gene fused to *TPM3* or to *TPM4*, [17, 18] two genes encoding for a non-muscle tropomyosin. Both TPM3-ALK and TPM4-ALK proteins cause constitutive autophosphorylation and activation of ALK [19] with consequent downstream activation of STAT3. Many other fusion proteins are found in IMT, including CLTC-ALK, ATIC-ALK, SEC31A-ALK, RANBP2-ALK, PPFIBP1- ALK, and CARS-ALK.

In 2007, the chromosomal rearrangement involving the *ALK* and *EML4 (echinoderm microtubule-associated protein like 4)* genes was identified in about 5% of NSCLC patients [19]; the rearrangement is frequently observed in relatively younger patients, non- or light smokers, and those with adenocarcinoma histology without other genetic disorders, such as mutations of the *epidermal growth factor receptor (EGFR)* gene [20, 21]. All 13 fusion variants of EML4-ALK contain exons 20–29 of *ALK*, which encode the entire intracellular segment of ALK, and 8 different *EML4* exons (2, 6, 13, 14, 15, 17, 18, and 20). Other ALK fusion proteins have also been described in NSCLC, including KIF5B-ALK, TFG-ALK, KLC1-ALK, PTPN3-ALK, and STRN-ALK with the consequent activation of downstream signalings including Ras/ERK1/2, PI3K/Akt, and JAK/STAT. Importantly, inhibitors of ALK significantly suppressed the growth of BA/F3 cells that express EML4-ALK [22] thus identifing *ALK* rearrangements as new potential therapeutic targets. Although the proportion of NSCLCs with EML4- ALK fusion proteins is low (5%), the absolute number results high as a consequence of the relatively high incidence of NSCLC. Therefore, ALK-rearranged NSCLC cases represent the largest population amenable of therapy with ALK inhibitors than other known ALK-related cancers combined.

Despite the variety of ALK fusion partners, some common features can be highlighted. Whenever an ALK fusion occurs, it will result in the activation of the ALK protein kinase domain that plays a key role in the tumorigenic process. The partner protein, which is the C-terminal of the fusion protein, controls the protein’s behavior, such as expression level and activation. Therefore, these cells uncontrolledly proliferate, survive, differentiate, and migrate, consequently leading to cancer [23].

Indeed, the initiation of transcription of ALK fusion proteins is driven by the regulatory regions of the partner gene; the subcellular localization of the fusion protein is determined by the partner protein, which means that ALK activity can occur in the nucleus and/or in the cytoplasm. The dimerization of ALK fusions occurs through the ALK partner protein and involves trans-autophosphorylation, and thus activation of the ALK kinase domain.

However, gene fusions are only a part of the genetic alteration affecting ALK gene.

Amplification of the *ALK* locus and consequent overexpression of ALK protein has been reported in many different types of cancer cell lines and human tumor samples [16, 24] including melanoma, NSCLC, neuroblastoma, glioblastoma, rhabdomyosarcoma, ovarian cancer, breast cancer, astrocytoma, Ewing’s sarcoma, and retinoblastoma.

Regardless of amplification, ALK overexpression is widely observed in nearly 100% of basal cell carcinoma [25] and in more than 50% of neuroblastomas, with only 10% of primary neuroblastomas displaying also *ALK* gene amplification. On the other side, ALK mutation is found in 7% of sporadic neuroblastomas and 50% of familial neuroblastomas. Most of the ALK mutations described are located within the kinase domain, and several have been shown to behave oncogenetic in in vitro and in vivo models [24, 26, 27]. *ALK* point mutations have been found mainly in neuroblastoma, as well as in NSCLC and ATC (anaplastic thyroid cancer). NSCLC and IMT gateway mutations often occur as secondary mutations in the context of acquired resistance to specific inhibitors, such as crizotinib.

### Targeting ALK in cancer

The presence of ALK fusion proteins and the constitutive ALK tyrosine kinase activity represent a therapeutic target in all malignancies with *ALK* rearrangement. Further, considering that ALK is not widely expressed in adult tissue, few toxic effects might be expected from treatment aimed at blocking ALK function.

The first ALK inhibitor introduced in the treatment of ALK-dependent NSCLC has been crizotinib, a potent oral small-molecule tyrosine kinase inhibitor of ALK, as well as c-MET and C-ros oncogene 1 (ROS1) kinases. Early phase I studies with crizotinib in ALK-fusion-positive metastatic pre-treated NSCLC patients [28–30] showed an objective response rate (ORR) of 57%.

Two phase III studies, which led to the United State Food and Drug Administration (FDA) approval of crizotinib, further confirmed the superiority of crizotinib on standard chemotherapy as first or second line therapy of ALK-rearranged NSCLC patients [31, 32]. In the PROFILE 1007 study, crizotinib showed ORR of 65% as compared with 20% with either pemetrexed or docetaxel in patients who had failed one prior platinum-based regimen [31]. In treatment-naive ALK-positive NSCLC (PROFILE 1014), crizotinib significantly improved progression-free survival (PFS) (median, 10.9 months versus 7 months) and ORR as compared to standard first-line chemotherapy [32] indicating a clear and effective new strategy window for ALK-rearranged patients. Of particular interest, crizotinib was associated with disease control in patients with brain metastasis [33].

Similarly, crizotinib also showed therapeutic response in ALK-fusion-positive IMT patients [34] and pediatric patients with anaplastic large cell lymphoma and IMT [35].

However, some patients do not respond to crizotinib or even after an initial response, lasting a median of 12–13 months, acquired resistance occurs.

**Fig. 3:**
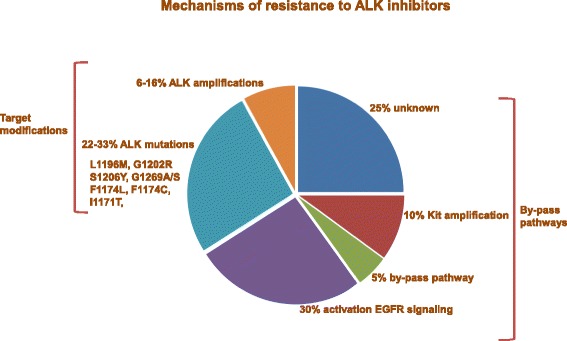
Mechanisms of resistance to ALK inhibitors. Resistance to ALK inhibitors may be mediated by acquired secondary mutations in the ALK kinase domain (F1174 L, F1174C, L1196 M, I1171T, G1202R, S1206Y, G1269S, and G1269A) or *ALK* gene amplification indicating the persistance of ALK dependency. Resistance can also be mediated by activation of alternative survival pathways such as the EGFR or the insulin-like growth factor pathways

Several resistance mechanisms have been described, mostly defined as ALK-dependent or non ALK-dependent according to the maintenance or not of the oncogenetic role of ALK signaling. Acquired secondary mutations in the ALK kinase domain (F1174 L, F1174C, L1196 M, I1171T, G1202R, S1206Y, G1269S, and G1269A) or *ALK* gene amplification [36–40] are known to be associated with resistance. Resistance can also be mediated by activation of alternative ALK-independent survival pathways such as the EGFR or the insulin-like growth factor pathways or the RAS/SRC and AKT/mTOR signalings [30–43] (Fig. 3).

The most common site of relapse after crizotinib treatment is the central nervous system (CNS) as a consequence of the to the P-glycoprotein (P-gp)-mediated efflux that is responsible for the poor accumulation of the drug in this site.

As previously mentioned, although the acquisition of resistance, most tumors progressing on crizotinib continue to depend on ALK signaling and are sensitive to more potent, structurally distinct, second-generation ALK inhibitors, such as ceritinib, alectinib, brigatinib, and lorlatinib.

In preclinical studies, ceritinib efficiently inhibited several *ALK* secondary mutations developed after crizotinib therapy [44]. In a phase I study, ceritinib was administered to 114 *ALK*-rearranged, crizotinib-naïve and -resistant NSCLC patients [45] achieving an ORR of 58%, and demonstrating activity also in those patients with *ALK* gene amplification or mutations (L1196 M, S1206Y) developed after crizotinib therapy. Ceritinib gained approval as the second-line treatment after crizotinib, thus expanding the tools of effective therapeutic options for ALK-positive NSCLC. The approval of ceritinib was based on the positive data in the ASCEND-2 and ASCEND-3 studies, in which an increased ORR was found in both crizotinib-naive and -resistant patients [46, 47]. In the ASCEND-4 study, progression-free survival (PFS) was 16.6 months compared to 8.1 months in the platinum-based chemotherapy arm [48].

Alectinib is a second generation potent and selective anti-ALK therapy able to bypass resistance to crizotinib exhibiting in vitro and in vivo activity in several *ALK*-resistant mutations, including L1196 M, F1174 L, R1275Q, and C1156Y [49, 50].

In 2016, alectinib gained the FDA and European Medicines Agency (EMA) approval as a second-line therapy in *ALK-*rearranged NSCLC patients treated with crizotinib [51].

Two phase I-II studies showed that alectinib was well tolerated. The first study (Japanese Phase I–II study (AF-0001JP), conducted in ALK inhibitor-naïve patients with *ALK*-rearranged NSCLC showed objective response of 93.5% [52]. Treatment was continued reaching a 3-year PFS of 62% (95% confidence interval [CI], 45%–75%) and a 3-year overall survival (OS) of 78%. Importantly of the 14 patients with brain metastases at baseline, six ones remained without progression [53]. The second study tested the efficacy of alectinib in patients with crizotinib-resistant *ALK*-rearranged NSCLC and showed objective response of 55% [54].

Then, two phase II studies (NP28761 and NP28673) in *ALK*-positive, crizotinib-resistant patients showed similar results with an ORR of 48% and 50% respectively and a median PFS of 8.1 months (95% CI, 6.2–12.6 months) and 8.9 months (95% CI, 5.6–11.3 months) respectively [55, 56]. Both studies also demonstrated the efficacy of alectinib against CNS metastasis. This can be explained by the increased penetration in the CNS, as alectinib is not transported by P-gp-mediated transport and thus reaches a higher CNS- to-plasma ratio than crizotinib [57].

The role of alectinib in the first line therapy of *ALK*-positive NSCLC patients has been explored in two phase III trials. The J-ALEX trial compared the efficacy and safety of alectinib versus crizotinib in japanese *ALK*-positive advanced or recurrent NSCLC patients with no prior ALK inhibition therapy. Patients receiving alectinib had not yet reached the median PFS, while patients receiving crizotinib showed a median PFS of 10.2 months. Safety profile was in favour of alectinib with grade 3–4 toxicities less frequent in the alectinib arm (27%) compared to the crizotinib arm (52%) [58].

In addition to the J-ALEX trial, the results from the ALEX phase III randomized clinical trial conducted in non-asian *ALK*-positive untreated patients comparing alectinib with crizotinib, have been recently presented. The authors reported similar results regarding the superiority of alectinib versus crizotinib: lower chance of progression (41% vs 68%), higher 12-month event-free survival rate (68.4% vs 48.7%), lower rate of CNS progression (12% vs 45%), higher response rate (82.9% vs 75.5%), and less adverse events (41% vs 50%) [59]. These results strongly support the role of alectinib as a first-line treatment instead of crizotinib.

These studies suggest that crizotinib-resistant tumors keep dependency on ALK signaling, and this is extremely interesting since both ceritinib and alectinib are able to inhibit *ALK*-positive NSCLC mutants harboring different resistance mutations [60].

Other ALK inhibitors are also in development such as entrectinib, lorlatinib and brigatinib, the latter received regulatory approval recently [61]. Among the various investigational drugs, entrectinib, (a multitarget drug, previously known as RXDX-101 and NMS-E628) has demonstrated a promising antitumor activity. It is a selective inhibitor of ALK, of the TPM A, B and C (encoded by the *NTRK1, 2, and 3* genes) and of ROS1, with a 36-fold greater potency than crizotinib [62, 63]. Two phase I trials of entrectinib ((ALKA-372-001 and STARTRK-1) had evaluated its safety profile, showing that entrectinib was well tolerated, with the majority of adverse event being reversible and grades 1–2. Confirmed responses were achieved in five different tumoral histologies in both adult and pediatric patients, including NSCLC, colorectal cancer, mammary analog secretory carcinoma, melanoma, and renal cell carcinoma, from 4 weeks after starting therapy and lasted until two years. Responses were observed in 19/24 (79%) patients with extracranial solid tumors and in two patients with brain tumors, thus confirming that entrectinib is highly CNS-penetrant. In particular, one patient with *NTRK* rearranged astrocytoma obtained tumor shrinkage and one patient with *SQSTM1-NTRK1*-rearranged lung cancer with multiple brain metastasis, not underwent to radiotherapy, achieved a complete CNS response with entrectinib [63]. Considering the high variability of study population, the secondary endpoints median PFS was 8.3 months in ALK positive patients, while it was not reached in *NTRK* and *ROS1* rearranged patients (3,6 and 6,5 months, respectively, as minimum value of CI, confidence interval, 95% to not reached) and median OS has not been reached in all subgroups, with 89.4% of patients alive at one year, after a median duration of follow up of 15 months [63]. These results of the phase I study of entrectinib in patients with *NTRK/ROS1/ALK* gene fusions have led to the initiation of an open-label, multicenter, global, phase II basket study (STARTRK-2, NCT02568267) to test the use of entrectinib in patients having tumors with these gene rearrangements, still ongoing.

Recently, data of phase I trial of lorlatanib in *ALK* or *ROS1* rearrangement positive patients was published [64]. Lorlatanib showed a very good tolerability profile with only one dose-limiting toxicity occurred at 200 mg and recommended phase II dose fixed to 100 mg once daily. Among *ALK* positive patients enrolled in this trial, 19/41 (46%) showed objective response, of which 11/19 (58%) had been treated previously with two or more lines of anti-ALK targeted agents, including patients with brain progression desease [64].

The actual stage of clinical development of ALK inhibitors and their specific targets are indicated in Table 1.

**Table 1:** Targets and phase of clinical development of ALK inhibitors

Drug	Targets	Phase of clinical development	References
Crizotinib	ALK c-MET ROS1	Indication worldwide for therapy of NSCLC harbouring ALK gene rearrangement in first and second line	• Shaw AT, et al. NEJM 2013 [32] • Solomon BJ, et al. NEJM 2014 [33]
Alectinib	ALK, including ALK secondary mutationsRET	- Approval from FDA (December 2015) and EMA (February 2017) in second line therapy of NSCLC harbouring ALK gene after progression to crizotinib - FDA granted fast approval in first line setting based on results of phase II and III clinical trials	• Larkins E, et al. CCR 2016 [52] • Shaw AT, et al. Lancet Oncol 2016 [56] • Hida T, et al. Lancet 2017 [59] • Peters S, et al. NEJM 2017 [60]
Ceritinib	ALK, including ALK secondary mutations IGF-1R ROS1 STK22D	- Recent fast approval from FDA in second line therapy of NSCLC harbouring ALK gene after progression to crizotinib (May 2017) based on results of phase II clinical trials	• Crinò L, et al. JCO 2016 [48] • Shaw AT, et al. JTO 2016 [47]
Brigatinib	ALK, including ALK secondary mutations ROS1 EGFR NTRK1	- Recent fast approval from FDA in second line therapy of NSCLC harbouring ALK gene after progression to crizotinib (April 2017) based on results of phase II clinical trials	• Markham A, et al. Drugs 2017 [61]
Lorlatanib	ALK and ROS1, including all known their mutant forms	Ongoing phase III trials in NSCLC first line versus crizotinib	• ClinicalTrials.gov, NCT03052608
Entrectinib	ALK, Including ALK secondary mutations NTRK1, 2, and 3 ROS1	Ongoing phase II basket trial STARTRK-2 (NCT02568267)	• Drilon A, et al. Cancer Discovery 2017 [63]

## Conclusions

So far, the ideal start and sequence of ALK inhibitors still need to be defined. The choice between different ALK inhibitors may depend on the *ALK* resistance mutations occurring during treatments. Each ALK inhibitor indeed exhibits its own molecular response, and continuous surveillance on resistance mutations is crucial for an effective treatment strategy. Depending on the type of crizotinib- resistant mutations, patients can now be offered the choice between two potent and effective ALK inhibitors, and other even more potent inhibitors are under clinical investigation, improving long-term treatment strategies [56, 65].

It seems evident from the recent success of ceritinib and the fast-track FDA approval of alectinib that genomic profiling of NSCLC tumors is necessary to personalize the treatment of *ALK*-positive lung cancer patients [65]. Especially after progression on second-generation ALK inhibitors, different mutations may occur. Entrectinib may have a role in this setting of patients; even if patients who had received crizotinib or other ALK-targeted drugs ceritinib or alectinib did not benefit from treatment in terms of responses in phase I trials with entrectinib, further investigation are needed to clarify the activity of entrectinib in ALK pre-treated patients, considering that it is active against resistance mutations such as the *ALK L1196 M* mutation, that can onset under crizotinib therapy, and that it is very CNS-penetrant [63]. Ongoing phase II trial of entrectinib is enrolling previously treated ALK positive NSCLC patients with only CNS progression disease.

In addition, possibility of rechallenging therapies cannot be excluded: in a recent report, Shaw et al. showed an interesting resensitization of a *ALK*-rearranged NSCLC patient being retreated with crizotinib. The patient, indeed, after acquisition of resistance to first-line crizotinib was treated with chemotherapy and then with second-generation ALK inhibitor ceritinib. As resistance to ceritinib occurred, lorlatinib, the third-generation ALK inhibitor, was administered. Once the patient became lorlatinib resistant and developed an L1198F mutation in ALK the patient was subsequently re-treated with crizotinib obtaining again disease remission [66]. Data from phase I trial of lorlatanib suggest a potential role of this drug after resistance to various anti-ALK agents, in *ALK* positive patients, including the subset of patients with brain metastasis [64]. Ongoing phase III trial of lorlatanib will clarify its activity in first line of therapy for *ALK* positive patients (NCT03052608).

In addition to keep ALK blocked with specific inhibitors, there are pharmacological strategies that allow for its indirect targeting. Specifically, inhibitiong heat-shock proteins (HSP), namely HSP90, a chaperone protein that stabilizes a wide variety of proteins, including ALK, has shown some preclinical efficacy in crizotinib-resistant *ALK* fusions (*EML4-ALK* and *NPM1-ALK*), including secondary resistant mutants in lung cancer models [67]. In addition, several drug combinations, including ALK inhibitors and other receptor tyrosine kinase inhibitor, such as Insulin-like growth factor 1 receptor-1 (IGF1R) [68], Mitogen-activated protein kinase kinase (MEK) [69–71] and HSP90 [67] inhibitors, are being explored in preclinical/clinical studies.

Immune-based therapeutic strategies are under investigation in *ALK*-positive ALCL. The evidence of ALK fusion protein as good immunogenic stimulus [16] is leading to several strategies for anti-ALK immune-based treatments of chemotherapy-resistant ALCL. Similarly, as recent preclinical data indicate, the immune checkpoint proteins are induced in *ALK*-positive NSCLC tumors [72], thus, combination therapies of checkpoint (PD-1/PD-L1, CTLA-4) and ALK inhibitors are being explored in the clinical setting for *ALK*-positive NSCLC patients (NCT02393625, NCT01998126).

Chemotherapy also remains a viable option in NSCLC patients with ALK translocations where pemetrexed-based chemotherapy may be more effective than other non-pemetrexed combinations [73].

## Abbreviations

aa: Aminoacids
ALCL: Anaplastic large cell lymphoma
ALK: Anaplastic lymphoma kinase
ATC: Anaplastic thyroid cancer
CI: Confidence Interval
CNS: Central nervous system
CR: Complete response
DLBCL: Diffuse large B-cell lymphoma
EGFR: Epidermal growth factor receptor
EMA: European Medicines Agency
EML4: echinoderm microtubule-associated protein like 4
FDA: Food and Drug Administration
HSP: Heat-shock proteins
IGF1R: Insulin-like growth factor 1 receptor-1
IMT: Inflammatory myofibroblastic tumors
JAK: Janus kinase
LTK: Leukocyte tyrosine kinase
MAPK: Mitogen-activated protein kinase
MEK: Mitogen-activated protein kinase kinase
MSN: Moesin
mTOR: Mammalian target of rapamycin
MYH9: Myosin 9
NPM: Nucleolar phosphoprotein B23 numatrin
NSCLC: Non-small cell lung cancer
ORR: Objective response rate
OS: Overall survival
PFS: Progression-free survival
PI3K: Phosphoinositide 3-kinase
PR: Partial response
PTPRB/PTPRZ1: Protein tyrosine phosphatase beta and zeta complex
ROS1: C-ros oncogene 1
RTK: Receptor tyrosine kinase
SD: Stable disease
SQSTM1: Sequestosome 1
STAT: Signal transducer and activator of transcription
TFG: TRK-fused gene
TPM3: Tropomyosin 3
TPM4: Tropomyosin 4
